# Histone deacetylase inhibitor during in vitro maturation decreases developmental capacity of bovine oocytes

**DOI:** 10.1371/journal.pone.0247518

**Published:** 2021-03-05

**Authors:** Thais Preisser Pontelo, Mauricio Machaim Franco, Taynan Stonoga Kawamoto, Felippe Manoel Costa Caixeta, Ligiane de Oliveira Leme, Nayara Ribeiro Kussano, Marcio Gilberto Zangeronimo, Margot Alves Nunes Dode

**Affiliations:** 1 Federal University of Lavras, Veterinary Science, Lavras, Minas Gerais, Brazil; 2 Federal University Uberlândia, Animal Science, Uberlândia, Minas Gerais, Brazil; 3 Institute of Genetics and Biochemistry of Federal, University of Uberlandia, Uberlândia, Minas Gerais, Brazil; 4 Embrapa Genetic Resources and Biotechnology, Brasília, Distrito Federal, Brazil; 5 University of Brasilia, Animal Science, Brasilia, Distrito Federal, Brazil; 6 Federal University of Espírito Santo, Animal Science, Vitória, Espírito Santo, Brazil; 7 University of Brasilia, Institute of Biology, Brasilia, Distrito Federal, Brazil; Justus Liebig Universitat Giessen, GERMANY

## Abstract

This study aimed to evaluate the effect of scriptaid during pre-maturation (PIVM) and/or maturation (IVM) on developmental competence of bovine oocytes. Cumulus-oocyte complexes (COCs) were submitted to PIVM for 6 h in the presence or absence of scriptaid. COCs were distributed into five groups: T1-IVM for 22 h, T2-PIVM for 6 h and IVM for 22 h, T3-PIVM with scriptaid for 6 h and IVM for 22 h, T4-PIVM for 6 h and IVM with scriptaid for 22 h, and T5-PIVM with scriptaid for 6 h and IVM with scriptaid for 22 h. Nuclear maturation, gene expression, cumulus cells (CCs) expansion, and embryo development and quality were evaluated. At the end of maturation, all groups presented the majority of oocytes in MII (P>0.05). Only *HAT1* gene was differentially expressed (P<0.01) in oocytes with different treatments. Regarding embryo development at D7, T4 (23%) and T5 (18%) had lower blastocyst rate (P<0.05) than the other treatments (T1 = 35%, T2 = 37% and T3 = 32%). No effect was observed when scriptaid in PIVM was used in less competent oocytes (P>0.05). In conclusion, presence of scriptaid in PIVM and/or IVM did not improve developmental competence or embryo quality.

## Introduction

In vitro maturation (IVM) is one of the most critical steps in the in vitro production of embryos (IVP) since oocytes are obtained from follicles of different sizes and constitute a very heterogeneous population [1–4]. Considering that after the removal of the oocytes from the follicular environment they spontaneously resume meiosis regardless their level of competence, many of the oocyte submitted to IVM are not fully competent yet and cannot support embryonic development [4,5]. One of the ways to overcome this problem is to use a pre-maturation (PIVM) period, which can provide oocytes an extra time to undergo changes such as additional transcripts stock formation, and cytoplasm reorganization [6–8].

A variety of agents that maintain high levels of cAMP and retain the oocyte at GV such as stimulators of adenylate cyclase or inhibitors of type 3 A phosphodiesterases (PDE3A) have been used for PIVM [6,7,9–16]. Even though the findings of all those studies are consistent regarding the effect of these agents on meiotic retention, their effect on the improvement of embryo development is contradictory. The main reason for these controversial results is that in most studies, the PIVM system consisted of only culture medium supplemented with a protein source, and a modulator of cAMP/cGMP. Moreover, the effect of PIVM use appears to vary according to the size of the follicles in which the oocytes were obtained. Zhang et al. [16] using NPPC (Natriuretic Peptide C) during PIVM in pig oocytes obtained from 3–8 mm follicles did not show any difference but when they were used only in oocytes from follicles of 3–4 mm, they observed higher blastocyst rates.

Among all the events needed to prepare the oocyte for fertilization, the post-translational histone modifications have important roles during oogenesis. Specifically, histone acetylation/deacetylation is essential to accumulate and store all the necessary mRNA, and to sustain remodeling of both maternal and paternal chromatin in the early embryo [17–19]. These histone modifications are established by two families of enzymes: histones acetyltransferases (HATs) and histone deacetylases (HDACs) [20–22]. A more acetylated pattern of histones induces a more open chromatin state, making it more accessible for transcriptional machinery, thereby allowing several transcription factors to have access to the gene promoters [23,24]. Differently, a more deacetylated pattern characterizes a more condensed chromatin, with transcriptional repression occurring in most genes [19].

In immature oocytes, at GV stage, histones are highly acetylated, but they lose their acetylation status as meiosis progresses. If fact, studies have shown that bovine oocytes showed higher levels of acetylation in the GV and in germinal vesicle break down (GVBD) stages compared to metaphase I (MI) and metaphase II (MII) stages, indicating that acetylation signals after GVBD practically disappear [25]. Therefore, if GV oocytes could be retained at prophase I and at the same time with histones in a hyperacetylated status after being removed from their follicles, they would keep transcription for a longer period increasing mRNA stock, and ultimately oocyte competence. Hyperacetylated status of histones can be induced by inhibitors of histone deacetylases (HDACis) such as trichostatin A, valproic acid, scriptaid, and sodium butyrate [26–28]. These inhibitors have been used mainly in embryo culture media to improve the development of clone embryo produced by somatic cell nuclear transfer (SCNT) [29–31]. Few studies reported the use of HDACi during maturation [32,33] however, when scriptaid was used during IVM of buffalo oocytes, the results showed an increased expression of genes related to acetylation, and improvement in the production of SCNT embryos [34].

Therefore, the aim of this study was to evaluate the use of scriptaid during pre-maturation and/or maturation stage in an attempt to improve oocyte competence, and *in vitro* development of bovine embryos.

## Material and methods

Unless otherwise indicated, chemicals were purchased from Sigma (St. Louis, MO, USA).

### Recovery and selection of oocytes

Ovaries from crossbred cows (*Bos taurus indicus* × *Bos taurus taurus*) were collected at Qualimax (Luziania GO) and Nippobras (Formosa, GO) slaughterhouses and transported to the laboratory in a 0.9% saline solution (NaCl 0.9%), supplemented with streptomycin sulphate (100 μg/ml) and penicillin G (100 IU/ml) at 35 to 36°C, with maximum transport time of 4 h. Cumulus–oocyte complexes (COCs) were aspirated from 3 to 8 mm diameter follicles with a syringe with 18-gauge needle and pooled in a 15 ml conical tube (TPP®, Trasadingen, Schaffhausen, Switzerland). After sedimentation, 10 ml of the supernatant follicle fluid was centrifuged for 5 min at 700 ×*g* and used for search and selection of COCs. Only COCs with four or more layers of cumulus cell (CC) and homogeneous cytoplasm were used for the experiment.

### Pre-maturation (PIVM)

Selected COCs were washed and transferred in number of 25 to 30 to a 150 μl drop of either PIVM or maturation medium covered with mineral oil, and then cultured for 6 h at 38.5°C in 5% CO_2_. PIVM medium consisted of TCM-199 with Earle’s salts (Gibco®, Invitrogen, Carlsbad, CA, USA) supplement with 0.075 mg/ml of amikacin, 0.2% free fatty acid albumin (BSA-FAF), 0.68 mM of L-glutamine, 1 mM of sodium pyruvate, 0.1 μM of cysteamine, 10^−4^ IU/ml recombinant follicle stimulating hormone [rFSH (Gonal-F®, Merck Serono, Rockland, MA, USA), and a meiotic inhibitor supplemented or not supplemented with scriptaid at the concentration of 500 nM [34]. The meiotic inhibitor used was NPPC at the concentration of 100 μM [14].

### Maturation *in vitro* (IVM)

After PIVM, 25 to 30 COCs were placed in 150 μl drops of maturation medium. The IVM medium consisted of TCM-199 Earl’s salts (Gibco ®) supplemented with 10% fetal bovine serum (FBS, Gibco ®), 0.01 IU/ml of follicle stimulating hormone (FSH), 0.1 of mg/ml L-glutamine, 0.075 mg/ml of amikacin, 0.1 μM/ml of cysteamine, and 0.2 mM of sodium pyruvate. The IVM was performed for 22 h at 38.5°C and 5% CO_2_ in air.

To assess meiotic stage, COCs were removed from the maturation medium at the beginning and end of PIVM (6 h) to verify meiotic arrest, and at the beginning and end of IVM (0 h and 22 h, respectively) to assess the reversibility of the arrest and progression of meiotic maturation. At each time point, they were washed in phosphate buffered saline (PBS), and denuded by repeated pipetting until CCs were completely removed. The oocytes were then fixed for 48 h in ethanol and acetic acid (3:1) and stained with 45% lacmoid in glacial acetic acid. The evaluation of meiotic stage was performed under a phase-contrast microscope (Nikon Eclipse E200, 1.000x), and oocytes were classified according to meiotic stages into: GV, GVBD, metaphase I (MI), anaphase I (AI), telophase I (TI), and metaphase II (MII). Any oocyte that has diffuse or undefined chromatin or has some chromosomal aberration was considered as degenerated or abnormal.

### Assessment of cumulus expansion

To determine CC expansion, each COC was measured after selection, and after PIVM and IVM using the Motic Images Plus 2.0 program (Motic China Group Co, Ltd., Xiamen, China). The CC expansion during PIVM and IVM was determined by the difference between the mean area of all COCs from each treatment before and after PIVM, and before and after IVM.

### *In vitro* embryo production (PIVE)

After IVM, COCs were transferred to 150 μl drops of fertilization medium, consisting of Tyrode’s through Albumin Lactate Pyruvate (TALP), supplemented with 0.5 mM/ml of penicillamine, 0.25 of mM hypotaurine, 25 mM of epinephrine, and 10 mg/ml of heparin. Frozen semen from the same bull that was previously tested for IVP of embryos, was used for all treatments and replicates. After thawing, the sperm were selected by Percoll gradient method (GE® Healthcare, Piscataway, NJ, USA) with 90% (400 μl) and 45% (400 μl) poured in 1.5 ml microcentrifuge tubes, and centrifuged for 5 min at 9000 rpm or 700 ×*g*. The resultant pellet was re-suspended and added into the fertilization medium in a final concentration of 1 × 10^6^ motile spermatozoa/ml. The oocytes were co-incubated with the sperm, for 18 to 20 h, at 38.5°C and 5% CO_2_ in air. The day of *in vitro* fertilization was considered as day 0 (D0). Eighteen hours after *in vitro* fertilization, the presumptive zygotes of all treatments were washed and transferred to 150 mL drops of synthetic oviduct fluid media containing amino acids, citrate, and inositol (SOFaaci [35]), supplemented with 0.4% of bovine serum albumin, and incubated at 38.5°C with 5% CO_2_ for 7 days. Embryos were evaluated on Day 2 (D2) for cleavage, and on Days 6–7 (D6-7) to determine the blastocyst rates.

### RT-qPCR

Total RNA was isolated from four pools of 20 oocytes for each treatment using RNeasy Plus Micro^®^ Kit (Qiagen, Hilden, Germany) according to the manufacturer’s instructions. Total RNA was used for cDNA synthesis using the First Strand cDNA Synthesis kit (Invitrogen Carlsbad, CA, USA) and the Oligo-dT (0.5 μg/μl) primers, according to the manufacturer’s instructions in a final volume of 40 μl. Reactions were incubated at 65°C for 5 min, 50°C for 5 min, and 85°C for 5 min.

qPCR analyses were performed using the 7500 Fast-Real-Time PCR System (Applied Biosystem, Foster City, CA, USA), and the Fast SYBR Green Master Mix^®^ Kit (Thermo Fischer). Each sample was analyzed in triplicate with amplification efficiency between 90 and 110%. The specificity of each PCR product was determined by analyzing the melting curve, and the amplicon size in agarose gel. The reactions were performed in a final volume of 25 μl using an equivalent of 0.62 oocytes per reaction. The qPCR conditions were 95°C for 5 min followed by 50 cycles of denaturation at 95°C for 10 s, and then annealing and extension at 60°C for 30 s. Histone acetyltransferase (*HAT1*), lysine acetyltransferase 2A (*KAT2A*), histone deacetylase 1 (*HDAC1*), histone deacetylase 2 (*HDAC2*), histone deacetylase 3 (*HDAC3*), and the histone deacetylase 8 (*HDAC8*) genes were analyzed. The primer sequences, fragment sizes, and annealing temperatures are listed in Table 1. The expression level of the 3 constitutive genes, including Glyceraldehyde 3-phosphate dehydrogenase (*GAPDH*), β-actin (*ACTB*), and peptidylprolyl isomerase A (*PPIA*) were analyzed using geNorm software [36], which indicated that PPIA gene was the most stable. This gene was used as a reference for data normalization. The relative expression of each gene was calculated using the ΔΔCt method with correction of the efficiency [37].

**Table 1:** Primer sequences, amplicon sizes in base pairs (bp), annealing temperatures, and reference GenBank accession numbers.

Genes	Sequence	Size (pb)	Temperature (°C)	GenBank
*HAT1*	F: AAT TGA GAG ACT TTG TGC TTG TGA	392	60	NM_001034347.1
	R: TTC AAT GAC ACG TCG ATA ATC TTC			
*HDAC1*	F: ATC GGT TAG GTT GCT TCA ATC TG	188	60	NM_001037444.2
	R: GTT GTA TGG AAG CTC ATT AGG GA			
*HDAC2*	F: TTC CTG GAA CAG GAG ACT TA	194	60	NM_001075146.1
	R: ATC ACC AGA TAG GGA GTC TG			
*HDAC3*	F: GAA GAG GCC ATT AGT GAA GAG	227	60	NM_001206243.1
	R: TCA GTC CTG TCG TAG GTT AG			
*HDAC8*	F: CCA AAG CAG TGG TCT TAC AGC	140	60	NM_001076231.2
	R: TCC TCC CAA GAT GAG CGT TG			
*KAT2A*	F: TGG GAT TTG CTT CCG CAT GTT TCC	81	60	XM_003587447.4
	R: TTG ACC TGC TCA TTG GAG GTG ACA			
*PPIA*	F: GGC GTG AAC CAC GAG AAG TAT AA	119	60	NM_178320.2
	R: CCC TCC ACG ATG CCA AAG T			

### Differential staining of embryos

Total cell number, trophectoderm (TE) cells, and inner cell mass (ICM) were stained using protocol described by Velazquez et al. [38] with slight modifications. Only expanded blastocysts (BX) at D7 of culture were used for differential cell staining using Hoeschst 33342 and propidium iodide (PI). Briefly, the BXs were washed 3 times in PBS supplemented with polyvinyl pyrrolidone (PVP-0.1%). Subsequently, the BXs were incubated in 500 μl of PI solution (PBS with 0.3% Triton X-100 and 100 μg/ml of PI solution) for 30 s. Blastocysts were washed 3 times in PBS/PVP, and then incubated in 500 μl of 4% paraformaldehyde fixation solution with 5 mg/ml Hoechst 33342 for 15 min. Finally, the embryos were washed with PBS/PVP, mounted on glass slide in a drop of glycerol, gently flattened with a coverslip, and visualized for cell counting. Cell counting was performed directly under a fluorescence microscope (Carl ZEISS Axioplan-2, LLC, United States) with UV excitation of 460 for Hoechst and 560 nm for PI. Thus, nuclei labeled with bisbenzimide appeared blue, and they were considered as ICM while the nuclei labeled with both bisbenzimide and PI appeared red and were classified as TE. Embryos with about 20–40% of ICM in relation to the number of total cells were considered of good quality.

## Experimental design

### Experiment 1: Effect of scriptaid during PIVM and/or IVM on nuclear maturation kinetics

This experiment aimed to determine if scriptaid could affect the progression of meiosis. Before performing the nuclear maturation kinetics, we evaluated how long scriptaid could be used during maturation without affecting oocyte developmental competence. Three different treatments (T) were administered as follows: T1) control; COCs were submitted to 22 h of IVM, T2) COCs were submitted to IVM with scriptaid for the first 6 h and the remaining 16 h without scriptaid, T3) COCs were submitted to IVM with scriptaid for 22 h. Based on the results, we chose to expose COCs to scriptaid for 22 h of IVM for subsequent experiments.

Then, we evaluated if COCs would remain in the GV stage after PIVM, and if they were able to resume and complete meiosis after being blocked for 6 h during PIVM. Five treatments were used: T1- IVM (control): COCs were matured for 22 h; T2- PIVM/IVM: COCs were submitted to 6 h of PIVM and 22 h of IVM; T3- PIVM + scriptaid/IVM: COCs were submitted to PIVM for 6 h in the presence of scriptaid and matured for 22 h; T4- PIVM/IVM + scriptaid: COCs were submitted to PIVM for 6 h and matured for 22 h in the presence of scriptaid; T5-PIVM + scriptaid/IVM + scriptaid: COCs were submitted to PIVM for 6 h in the presence of scriptaid and matured for 22 h, also in the presence of scriptaid.

To assess nuclear kinetic, COCs were collected and fixed at 0 h, after 6 h of PIVM with and without the addition of scriptaid, at 8 h and 22 h of IVM. After fixation, they were stained with lacmoid and evaluated for meiotic stage under a phase-contrast microscope. The control group was evaluated at 0 h, 8 h, 14 h and 22 h; we added an additional evaluation time (14 h) which corresponded to the 6 h of PIVM plus 8 h of IVM.

### Experiment 2: Effect of scriptaid during PIVM and/or IVM on transcript levels

The aim of this experiment was to analyze if the presence of scriptaid during PIVM and/or IVM could affect the expression profile of the genes involved in histone acetylation/deacetylation processes in bovine oocytes. Treatments were the same as those used in the previous experiment, being the oocytes of all treatment groups collected at 0 h and 6 h of PIVM, and 0 h and 22 h of IVM. For CCs removal, the COCs classified as grades 1 and 2 were transferred to a drop of 50 μl of saline in phosphate buffer (PBS), where the CCs were stripped out by successive pipetting until complete removal. After denudation, the oocytes were washed 3 times in PBS. The oocytes were transferred (2 μl) to a 0.2 ml tube, and twice the volume of RNAlater (4 μl) (Ambion, Life Technologies, Carlsbad, CA, USA) was added to the tube. These oocytes were stored in -80°C freezer until the day of qT-PCR analysis.

### Experiment 3: Effect of scriptaid during PIVM and/or IVM on developmental competence of bovine oocytes

The objective of this experiment was to evaluate if the use of PIVM with or without the presence of HDACi would improve oocyte competence and embryo development. For this experiment oocytes obtained from 3–8 mm follicles were distributes into 5 treatments, which were same as those described in experiment 2. For all treatments, CCs expansion was evaluated after selection, and after pre-maturation and maturation phases. After maturation, the COCs were submitted to IVF and then cultured until D 7 of development. Embryos were evaluated on D2 for cleavage and on D6 and D7 for blastocyst rates. To evaluate the embryo quality, the expanded blastocyst (Bx) at D7 were stained by differential staining technique, that provides information about the total cell number, trophoectoderma cells (TE), inner cell mas (ICM), as well as the ratio of cell mass to total cells (ICM/total cells).

### Experiment 4: Effect of the HDAC inhibitor, scriptaid, on less competent oocytes

Considering that, it has been reported that PIVM may be more beneficial for the oocytes with lower developmental competence [10], we also evaluated whether scriptaid added to PIVM medium could improve oocyte competence. We chose to use less competent oocytes obtained from 1–3 mm follicles [39], which were evaluated for embryo production and embryo quality. Follicles of 1–3 mm diameters were dissected from the ovarian cortex, classified, selected morphologically and measured. Oocytes obtained from dissected follicles were divided into 3 treatment groups: 22 h IVM, PIVM + 22 h IVM, and PIVM with scriptaid + 22 h IVM. Oocytes of 3–8 mm from aspirated follicles were used as controls and submitted to the same treatments. Cleavage rate on D2, blastocyst rate on D6 and D7, and embryonic developmental speed were evaluated. At D7, expanded blastocysts Bx embryos were stained with differential staining method.

## Statistical analyses

Data on nuclear maturation, embryonic development, and proportion of ICM/total cells were analyzed using chi-square test (P < 0.05). The comparison between treatment groups regarding cumulus cell expansion, gene expression, number of TE cell, number of ICM cells, and total cell number was performed by analysis of variance (ANOVA) followed by Tukey’s test at significance level of P < 0.05. Non-parametric data of gene expression and the number of TE and ICM cells were analyzed using the Kruskall Wallis test. All analyses were performed by GraphPad Prism version 7 for Windows (GraphPad Software, La Jolla California USA).

## Results

### Experiment 1: Effect of scriptaid during PIVM and/or IVM on nuclear maturation kinetics

Initially we evaluated how long scriptaid could be used during maturation without affecting oocyte developmental competence. Cleavage rate (D2) was similar (P > 0.05) between the control (82.1%; n = 179) and the groups in which scriptaid was administered for 6 h (77.7%; n = 175) or 22 h (77.0%; n = 196) of maturation. However, blastocyst rate on D7 was lower (P < 0.05) in groups with scriptaid treatment for the first 6 h (17.7%) of IVM than in the control group (27.95%) and IVM treatment group with scriptaid for 22 h (30.6%) (S1 Table). Then, the use of scriptaid for 22 h was chosen for the remaining experiments.

To determine if scriptaid could affect meiosis progression, nuclear maturation kinetics was evaluated. The percentage of oocytes at GV stage was similar (P > 0.05) among the control and treatment groups in which COCs were submitted to PIVM, either in the presence or absence of scriptaid (Table 2). As for meiosis progression, after 8 h of IVM the control group presented majority of the oocytes at GVBD and GV stages, while treatment groups submitted to PIVM had majority of the oocytes in MI, AI, and TI phases. However, at the end of maturation, no differences between treatment groups was observed (P > 0.05), having all groups presenting around 90% of the oocytes at MII stage (Table 3).

**Table 2:** Evaluation of meiotic retention of bovine oocytes for 6 hours using pre-maturation (PIVM) for 6 h with 100 nM Type C natriuretic peptide added with 500nM scriptaid (Scrip).

Treatment	N	Stages of Maturation	
		GV	GVBD
Control 0h	69	66 (95.7%)	3(4.3%)
6h PIVM	54	47(87.0%)	7(12.9%)
6h PIVM c/Scrip	48	41(85.4%)	7(14.6%)

No significant difference were identified by Chi-Square test (P>0.05).

GV, germinal vesicle; GVBD, germinal vesicle breakdown.

**Table 3:** Meiotic stage at 8, 14 and 22 h of maturation of oocytes from the control group (no prematuration and no scriptaid) and of oocytes that had been submitted to a pre-maturation (PIVM) for 6 h with 100nM of Type C natriuretic peptide added or not of 500nM scriptaid (Scrip) and then were matured (IVM) in medium supplemented with or without 500nM scriptaid.

Treatment	N	Stages of Maturation			
		GV	GVBD	MI, AI, TI	MII
Control 8h IVM	73	21(28.8%)^a^	41(56.2%)^a^	11(15.1%)^d^	0(0)^b^
Control 14h IVM	42	0(0)^b^^c^	0(0)^d^	42(100%)^a^	0(0)^b^
6h PIVM + 8h IVM	44	0(0)^b^^c^	7(15.9%)^b^^c^	37(84.09%)^b^	0(0)^b^
6h PIVM c/Scrip + 8h IVM	49	0(0)^b^^c^	5(10.2%)^c^	44(89.7%)^b^	0(0)^b^
6h PIVM + 8h IVM c/Scrip	45	1(2.2%)^b^^c^	4(8.9%)^c^	40(88.9%)^b^	0(0)^b^
6h PIVM c/Scrip + 8h IVM c/Scrip	67	5(7.5%)^c^	20(29.9%)^b^	42(62.7%)^c^	0(0)b
Control 22h IVM	57	0(0)^b^	0(0)^d^	2(3.5%)^e^	55(96.49%)^a^
6h PIVM + 22h IVM	48	0(0)^b^^c^	0(0)^d^	2(4.2%)^de^	46(95.8%)^a^
6h PIVM c/Scrip + 22h IVM	52	0(0)^b^	0(0)^d^	4(7.7%)^de^	48(92.3%)^a^
6h PIVM + 22h IVM c/Scrip	54	0(0)^b^	0(0)^d^	2(3.7%)^e^	52(96.3%)^a^
6h PIVM c/Scrip + 22h IVM c/Scrip	53	0(0)^b^	0(0)^d^	4(7.5%)^de^	47(88.7%)^a^

^a,b,c^ Different letters in the same column indicate significant difference by Chi-Square test (P <0.05).

GV, germinal vesicle; GVBD, germinal vesicle breakdown.

### Experiment 2 effect of scriptaid during PIVM and/or IVM on transcript levels

**Fig 1:**
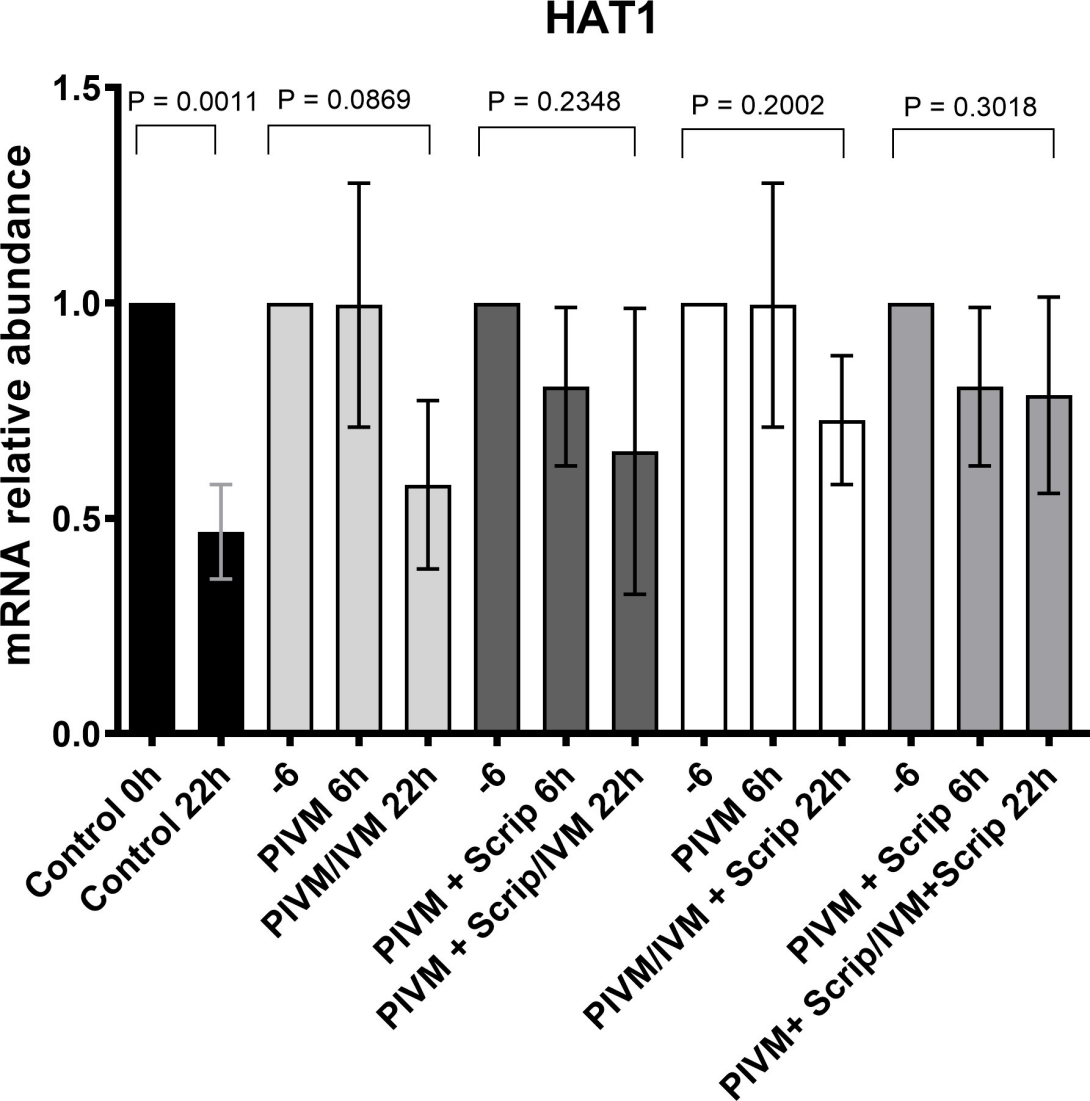
Transcripts levels of *HAT1* quantified by RT-PCR of bovine 20 oocytes in different treatment groups. The data (mean ± SD) were normalized using the formula ΔΔCT (Pfaffl, 2001) [37], and PPIA was the endogenous control. When the treatments were submitted to pre-maturation, we used the -6 legend to represent the control group 0 h. PIVM 6 h corresponds to oocytes that were submitted to pre-maturation for 6 h, PIVM + Scrip 6 h corresponds to oocytes submitted to pre-maturation with scriptaid for 6 h, PIVM/IVM 22 h corresponds to the oocytes that were pre-maturated and later matured in IVM medium for 22 h, PIVM + Scrip/22 h IVM corresponded to oocytes submitted to pre-maturation with scriptaid and then matured for 22 h, PIVM + Scrip/IVM + Scrip corresponds to oocytes that underwent pre-maturation and maturation with addition of scriptaid.

The expression profile of 6 genes involved in histone acetylation/deacetylation (*HAT1*, *KAT2A*, *HDAC1*, *HDAC2*, *HDAC3*, *HDAC8*) was analyzed in oocytes during PIVM and/or IVM with or without scriptaid. Of the 6 genes analyzed, only *HAT1* was differentially expressed among the treatment groups (Figs 1 and S2). While the expression of *HAT1* decreased after maturation relative to the expression before maturation (0 h) in the control group (P < 0.05), and in the group submitted to PIVM and IVM without scriptaid, the expression of *HAT1* in all other treatments with or without scriptaid at some point did not change during maturation (P > 0.05) (Fig 1).

### Experiment 3: Effect of scriptaid during PIVM and/or IVM on developmental competence of bovine oocytes

**Fig 2:**
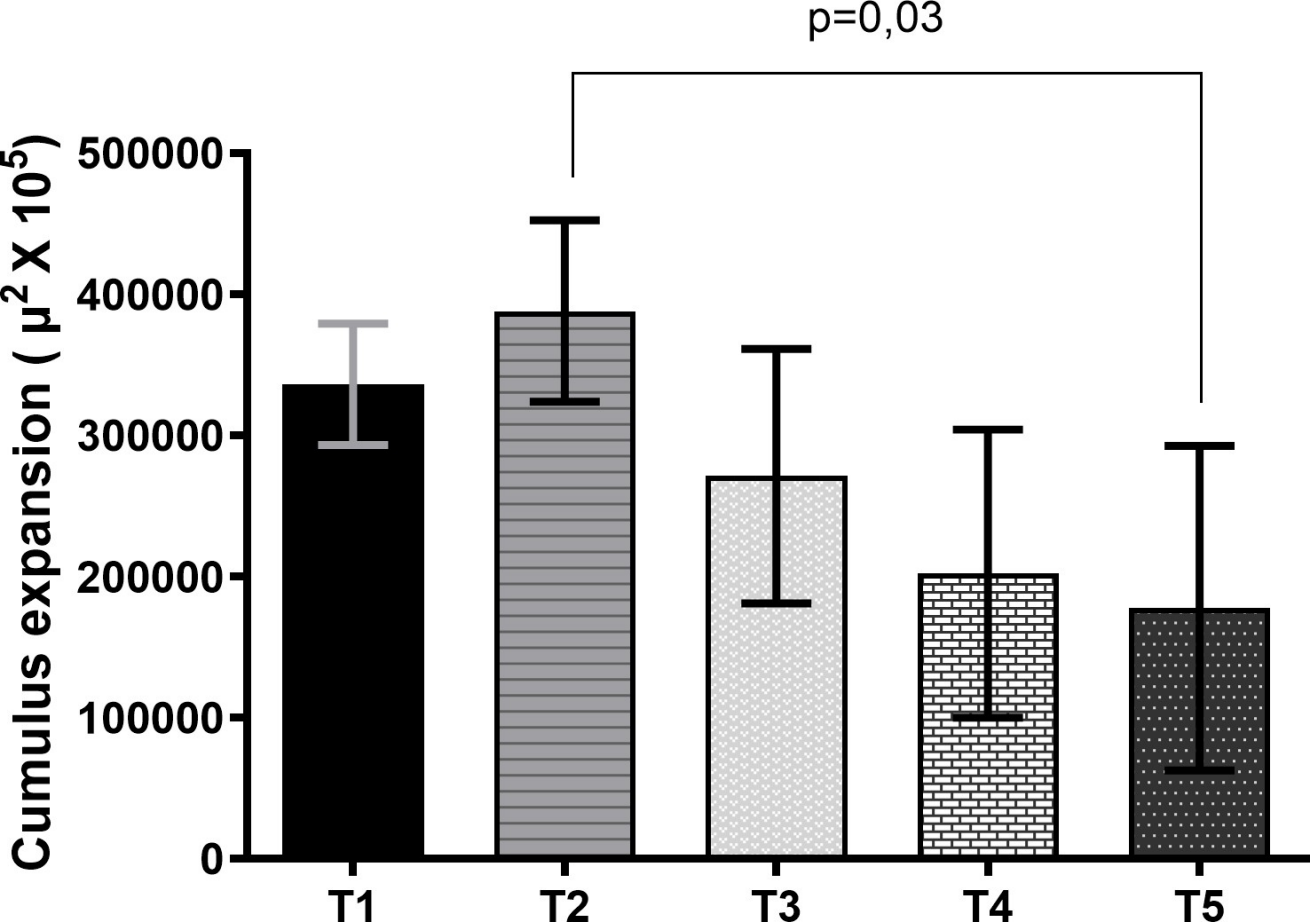
Expansion area of cumulus-oocyte complexes submitted to maturation (IVM) for 22 h (T1), pre-maturation (PIVM) for 6 h plus IVM for 22 h (T2), PIVM with scriptaid for 6 h plus IVM 22 h (T3), PIVM for 6 h plus IVM with scriptaid for 22 h (T4), PIVM for 6 h with scriptaid plus IVM 22 h with scriptaid (T5). Data are expressed as mean ± standard deviation and were analyzed by ANOVA and then compared by the Tukey test at the 0.05 level of significance. The data were obtained from the value of the initial mean area (0 hour) of the cumulus-oocyte-complexes (CCOs) subtracted from the value of the final mean area of the CCOs after IVM (22 h).

First, it was evaluated if scriptaid affected cumulus cells expansion after PIVM and IVM (S1 Fig). The data showed that there was no expansion of cumulus cells after PIVM. After 22 h of IVM, the treatment group that received scriptaid during PIVM and IVM presented a lower expansion of cumulus cells than PIVM and IVM treatment group without scriptaid (Fig 2). Regarding embryo development, the control group (T1) had a higher cleavage rate (P<0.05) compared to the other groups (Table 4). At D7, the treatments that received scriptaid in IVM, regardless of the use of scriptaid in PIVM showed a lower rate of blastocysts (P <0 .05) that the other groups.

**Table 4:** Effect of addition of scriptaid on PIVM and/or IVM on the rate of cleavage (D2) and rate of blastocysts in D6 and D7 on development of bovine embryos produced *in vitro*.

Treatments	N	Cleaved	Blastocyst D6	Blastocyst D7
T1	236	202(85.6%)^a^	24(10.2%)^a^	82(34.7%)^a^
T2	190	145(76.3%)^b^	12(6.3%)^a^	71(37.4%)^a^
T3	203	154(75.9%)^b^	3(1.5%)^b^	65(32.0%)^a^
T4	207	152(73.4%)^b^	2(1.0)^b^	47(22.7%)^b^
T5	177	136(76.8%)^b^	3(1.7%)^b^	32(18.1%)^b^

^a,b,c^ Different letters in the same column indicate significant difference by Chi-Square test (P<0.05).

T1: Cumulus-oocyte complexes (COCs) submitted to maturation (IVM) for 22 h.

T2: COCs submitted to pre-maturation (PIVM) for 6 h plus IVM for 22 h.

T3: COCs submitted to PIVM with scriptaid for 6 h plus IVM 22 h.

T4: COCs submitted to PIVM plus IVM with Scriptaid for 22 h.

T5: COCs submitted to PIVM with scriptaid plus IVM with scriptaid for 22 h.

Only the treatment group in which scriptaid was given during PIVM and IVM had lower number of total cells than the control (P < 0.05) (Table 5). To evaluate the allocation of ICM and TE cells, the expanded blastocysts evaluated were divided into 3 groups: 0%-20%, 20%-40%, and 40%-60% ICM/total cells. The group that received scriptaid during PIVM and IVM showed a higher (P < 0.05) percentage of embryos between 0%-20% (17%, 5/30), and a lower (P < 0.05) percentage of embryos between 20%-40% (67%, 20/30) than the control (2,6%, 1/39; 87.1%, 34/39, respectively) (S2 Table).

**Table 5:** Effect of the addition of 500nM of scriptaid during pre-maturation (PIVM) and/or maturation (IVM) on the amount of cells of the internal cell mass (ICM) and trophoectoderma (TE).

Treatment	N	Total Cells	TE	%TE	ICM	%ICM
T1	39	192.59^a^	133.10	69.04	59.49	30.96
T2	39	178.79^a^^b^	121.67	67.93	57.13	32.07
T3	32	189.53^a^	133.31	74.54	56.22	29.53
T4	30	178.10^a^^b^	124.60	73.91	53.50	30.30
T5	30	161.53^b^	113.67	69.99	47.87	30.01

^a,b,c^ Different letters in the same column indicate significant difference by ANOVA (P<0.05). When the data did not follow normality were analyzed by Kruskall Wallis.

T1: Cumulus-oocyte complexes (COCs) submitted to maturation (IVM) for 22 h.

T2: COCs submitted to pre-maturation (PIVM) for 6 h plus IVM for 22 h.

T3: COCs submitted to PIVM with scriptaid for 6 h plus IVM 22 h.

T4: COCs submitted to PIVM plus IVM with Scriptaid for 22 h.

T5: COCs submitted to PIVM with scriptaid plus IVM with scriptaid for 22 h.

### Experiment 4: Effect of HDAC inhibitor, scriptaid, on less competent oocytes

Less competent oocytes obtained from 1–3 mm follicles submitted to PIMV with or without scriptaid showed a lower (P < 0.05) cleavage and blastocyst rate than oocytes obtained from larger follicles (Table 6). Regarding the amount of ICM, TE and total cells, all treatments were similar (P > 0.05) (Table 7).

**Table 6:** Effect of addition of scriptaid on pre-maturation (PIVM) of less competent oocytes (obtained from 1–3 mm follicles) on the rate of cleavage (D2) and rate of blastocysts in D6 and D7 on development of bovine embryos produced *in vitro*.

Treatments	N	Cleavage	Blastocyst D6	Blastocyst D7
T1	100	83(83,0%)^a^	13(13,0%)^a^	34(34,0%)^a^
T2	71	50(70,4%)^a^^b^	2(2,8%)^b^	13(18,3%)^b^^c^
T3	104	83(79,8%)^a^	4(3,8%)^b^	34(32,7%)^a^
T4	114	83(72,8%)^a^	3(2,6%)^b^	35(30,7%)^a^^c^
T5	63	32(50,8%)^b^	1(1,6%)^b^	8(12,7%)^b^
T6	67	39(58,2%)^b^	1(1,5%)^b^	9(13,4%)^b^

^a,b,c^ Different letters in the same column indicate significant difference by Chi-Square test (P <0.05).

T1: Cumulus-oocyte complexes (COCs) obtained from follicles of 3-8mm submitted to maturation (IVM) for 22 h.

T2: COCs obtained from follicles of 1-3mm submitted to IVM for 22 h.

T3: COCs obtained from of 3-8mm submitted to PIVM plus IVM 22 h.

T4: COCs submitted from of 3-8mm submitted to PIVM with scriptaid plus IVM 22 h.

T5: COCs submitted from of 1-3mm submitted to PIVM plus IVM 22 h.

T6: COCs submitted from of 1-3mm submitted to PIVM with scriptaid plus IVM 22 h.

**Table 7:** Effect of the addition of scriptaid during prematuration (PIVM) of less competent oocytes (obtained from 1–3 mm follicles) on the amount of cells of the internal cell mass (ICM) and trophoectoderma (TE).

Treatment	N	Total cells	TE	%TE	ICM	%ICM
T1	19	131	88	70	35	30
T2	17	136	93	77	34	23
T3	21	122	82	62	35	38
T4	19	140	96	71	35	28
T5	7	115	79	70	46	30
T6	14	122	81	74	39	25

No significant difference were identified by ANOVA (P<0.05).

T1: Cumulus-oocyte complexes (COCs) obtained from follicles of 3-8mm submitted to maturation (IVM) for 22 h.

T2: COCs obtained from follicles of 1-3mm submitted to IVM for 22 h.

T3: COCs obtained from of 3-8mm submitted to PIVM plus IVM 22 h.

T4: COCs submitted from of 3-8mm submitted to PIVM with scriptaid plus IVM 22 h.

T5: COCs submitted from of 1-3mm submitted to PIVM plus IVM 22 h.

T6: COCs submitted from of 1-3mm submitted to PIVM with scriptaid plus IVM 22 h.

As in the previous experiment, the cell allocation was analyzed. On the control group, the majority of the embryos belonged to 20%-40% ICM/Total category while no embryo was classified as 0%-20% category. The other treatment groups were similar to the control group, except the low competent oocytes not exposed to scriptaid, which showed a lower (P < 0.05) percentage of embryos classified as 20%-40% ICM/Total cell than the control (S3 Table).

## Discussion

Considering that low developmental competence of *in vitro* matured oocytes is related to incomplete cytoplasmic maturation due to their early removal from the follicles [2,40], it can be assumed that the use of PIVM may provide the oocytes with extra time to increase their competence [5,10,11]. One of the factors responsible for full competence is the accumulation of transcripts. It is well known that this accumulation is controlled by epigenetic modifications that regulate gene expression and the conformational state of chromatin. Among these epigenetic modifications, histone acetylation is one of the mark controlling the opening and closing of chromatin, and consequently regulating transcription [17,41,42]. Thus, we hypothesized that the addition of HDACi during PIVM and/or IVM could prevent histones deacetylation allowing additional transcription, and the accumulation of mRNA.

Among the HDACi available we chose to use scriptaid because it has been used with success in the production of clone embryo [34,43–46], and specially due to its low toxicity [47]. Because there is no information regarding the use of scriptaid during maturation of bovine oocytes, we did a pre-experiment aiming to define how long scriptaid in culture should be kept. Our results showed that blastocyst rate at D6 and D7 was lower when scriptaid was added during 6 h of IVM than when it was added during 22 h of IVM. Therefore, we chose to use 22 h for all the experiments.

Because it is well known that meiotic progression is affected not only by HDACis [32,33,48,49] but also by meiotic arrest agents [7,50] we evaluated the nuclear kinetics of the oocytes exposed to NPPC and scriptaid. The results showed that regardless of the addition of scriptaid, meiosis was retained during 6 h of PIVM. However, after the oocytes were removed from PIVM and placed in IVM, an acceleration of meiotic progression was observed in all groups compared to the control. We are not sure why meiosis progression was accelerated after meiotic block, but it is probable an effect of the blockage itself since it has also been reported in previous studies [51–53]. Moreover, no effect of the arrest or the presence of HDACi, on nuclear maturation rate was observed. Similar results were reported using trichostatin on other species such as mice and porcine during IVM [54,55].

Furthermore, we tried to find out if the presence of scriptaid during PIVM and/or IVM could affect gene expression pattern of acetylation related genes. From all the genes analyzed (*HAT1*, *KAT2A*, *HDAC1*, *HDAC2*, *HDAC3*, *and HDAC8*), only *HAT1* showed a difference in the expression pattern. Transcript levels of *HAT1* decreased during maturation in the control group (P = 0.001), suggesting that histone acetylation is reducing throughout maturation and that global deacetylation is progressing until MII is reached. [25,49,55–57]. It is interesting to note that treatments with scriptaid at any time (PIVM or IVM) showed no difference in the expression of any evaluated gene, suggesting that the acetylation may remain unchanged until the stage of MII owing to the use of scriptaid. In agreement with this, similar results were reported by Sun et al. [34], who used the same concentration of scriptaid in IVM of buffalo oocytes and found that it induced an increase in the expression of acetylation related genes in MII, including *HAT1*. Although we were expecting to see a change on the expression of genes besides *HAT1*, some studies have shown that treatments with HDACis may influence a small percentage of genes [58,59].

To evaluate the effect of PIVM with or without scriptaid on the oocyte developmental competence, embryo production and quality were assessed. According to our results, the use of PIVM with or without scriptaid did not affect embryo production on D7. However, when scriptaid was used in IVM a detrimental effect on embryo production was observed. If scriptaid is maintaining the levels of *HAT1*, it is possible that the prolonged use of the scriptaid allowed histones to remain acetylated until the end of maturation, inducing disorganization of the genome, and impairing subsequent embryonic development. It is well known that at the end of maturation, chromatin condensation associated with gene silencing [60,61] occurs. Therefore, an acetylation in MII would be detrimental to the oocyte and subsequent embryo development [18,54,57]. Besides embryo production, we also evaluated embryo quality using total cell number, and the proportion of ICM/total cells by differential staining [29,62,63]. Treatment with scriptaid during PIVM and IVM showed embryo with lower quality than the control group. The lower quality of embryos can be observed by the lowest cell number and by the lowest percentage of embryos with proportion of 20%-40% of ICM/total cells [64]. The results suggest that prolonged use of scriptaid may have affected the oocyte and, subsequently embryo quality.

As CCs play an important role in maturation, and undergo extensive changes during this period, we evaluated their expansion before and after 6 h of PIVM and 22 h of IVM. There was no expansion of the CCs observed after PIVM in any of the treatments, which supports the idea that meiosis was arrested during the 6 h. Lack of CCs expansion during PIVM period has also been reported in previous studies [15,65] but the effect of PIVM in CCs expansion after IVM is contradictory [65,66]. In our study, after maturation, only the group that received scriptaid during PIVM and IVM presented less expansion than PIVM and IVM groups without scriptaid. This same treatment also presented a later meiotic progression in relation to the other groups. This suggests that the use of scriptaid in both moments could be detrimental to maturation. As already mentioned, the prolonged exposure of the oocytes to scriptaid (PIVM and IVM) may have maintained high level of histone acetylation until the end of maturation. This is not desirable because in MII, the chromatin is poorly acetylated, and needs to be fully closed.

Some studies have shown an improvement in the developmental competence of the oocytes from small follicles [5,67,68] when PIVM was used. Then we decided to evaluate the use of scriptaid during PIVM of the oocytes obtained from follicles of 1–3 mm, which is considered less competent than the follicles > 3.0 mm [39,69,70]. As expected, the less competent group presented lower embryo production in relation to the control, which confirms the lower competence of this oocyte. Moreover, PIVM and the addition of scriptaid during PIVM did not improve embryos production. Our results suggest that a possible state of hyperacetylation provoked by HDACis prior to the resumption of meiosis is not necessary for acquiring oocyte competence.

According to our results, the use of scriptaid may have maintained the patterns of histone acetylation due to its effect on *HAT1* transcripts levels. However, the use of scriptaid during PIVM and IVM proved to be detrimental to embryonic development, and did not improve embryo development even in the less competent oocytes. Therefore, we do not recommend the use of scriptaid at these concentrations during PIVM because it would add another manipulation with no beneficial effect.

## Supporting information

S1 Fig Representative image illustrating the cumulus cell expansion measurements using the program Motic Image Plus 2.0 before pre-maturation (PIVM) (A) and after 22 h of maturation (B).
(PDF)

S2 Fig Transcripts levels of *KAT2A*, *HDAC1*, *HDAC2*, *HDAC3* and *HDAC8* quantified by RT-PCR of bovine 20 oocytes in different treatment groups. The data (mean ± SD) were normalized using the formula ΔΔCT (Pfaffl, 2001) [37], and PPIA was the endogenous control. When the treatments were submitted to pre-maturation (PIVM), we used the -6 legend to represent the control group 0 h. PIVM 6 h corresponds to oocytes that were submitted to pre-maturation for 6 h, PIVM + Scrip 6 h corresponds to oocytes submitted to pre-maturation with scriptaid for 6 h, PIVM/IVM 22 h corresponds to the oocytes that were pre-maturated and later matured in IVM medium for 22 h, PIVM + Scrip/22 h IVM corresponded to oocytes submitted to pre-maturation with scriptaid and then matured for 22 h, PIVM + Scrip/IVM + Scrip corresponds to oocytes that underwent pre-maturation and maturation with addition of scriptaid.
(PDF)

S1 Table Effect of adding scriptaid during 6 and 22 hours of in vitro maturation (IVM) on cleavage at day 2 (D2) and blastocysts rates at D6 and D7 of development. (PDF)

S2 Table Effect of scriptaid during pre-maturation and/or in vitro maturation on the proportion of cells of the internal cell mass relative to the amount of total cells (ICM/total cells). (PDF)

S3 Table Effect of scriptaid during pre-maturation (PIVM) of less competent oocytes (obtained from 1–3 mm follicles) on the amount of cells of the internal cell mass (ICM) and trophoectoderma (TE). (PDF)
