# Supplementary material for: Histone deacetylase inhibitor during in vitro maturation decreases developmental capacity of bovine oocytes
Source: PLoS One. 2021 Mar 5;16(3):e0247518. doi: 10.1371/journal.pone.0247518 (PMC7935280; doi:10.1371/journal.pone.0247518)
Supplement: S1 Fig — Representative image illustrating the cumulus cell expansion measurements using the program Motic Image Plus 2.0 before pre-maturation (PIVM) (A) and after 22 h of maturation (B). (PDF) [file pone.0247518.s001.pdf]

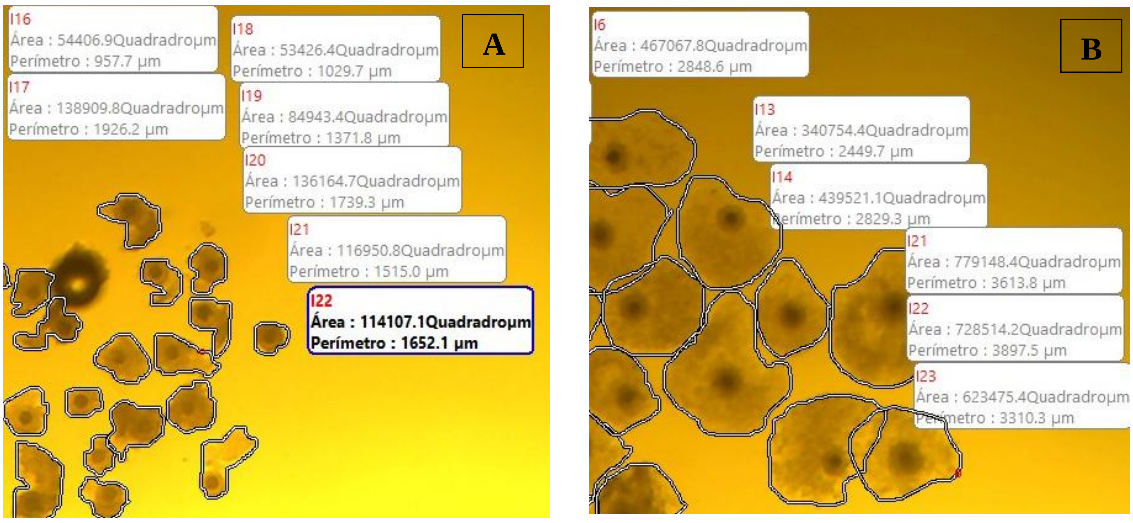

Fig. S1 Representative image illustrating the cumulus cell expansion measurements using the program Motic Image Plus 2.0 before the period of meiotic blockage considered as pre-maturation (PIVM) (A) and after 22 h of maturation (B).
