# Supplementary material for: Histone deacetylase inhibitor during in vitro maturation decreases developmental capacity of bovine oocytes
Source: PLoS One. 2021 Mar 5;16(3):e0247518. doi: 10.1371/journal.pone.0247518 (PMC7935280; doi:10.1371/journal.pone.0247518)
Supplement: S2 Fig — The data (mean ± SD) were normalized using the formula ΔΔCT (Pfaffl, 2001) [37], and PPIA was the endogenous control. When the treatments were submitted to pre-maturation (PIVM), we used the -6 legend to represent the control group 0 h. PIVM 6 h corresponds to oocytes that were submitted to pre-maturation for 6 h, PIVM + Scrip 6 h corresponds to oocytes submitted to pre-maturation with scriptaid for 6 h, PIVM/IVM 22 h corresponds to the oocytes that were pre-maturated and later matured in IVM medium for 22 h, PIVM + Scrip/22 h IVM corresponded to oocytes submitted to pre-maturation with scriptaid and then matured for 22 h, PIVM + Scrip/IVM + Scrip corresponds to oocytes that underwent pre-maturation and maturation with addition of scriptaid. (PDF) [file pone.0247518.s002.pdf]

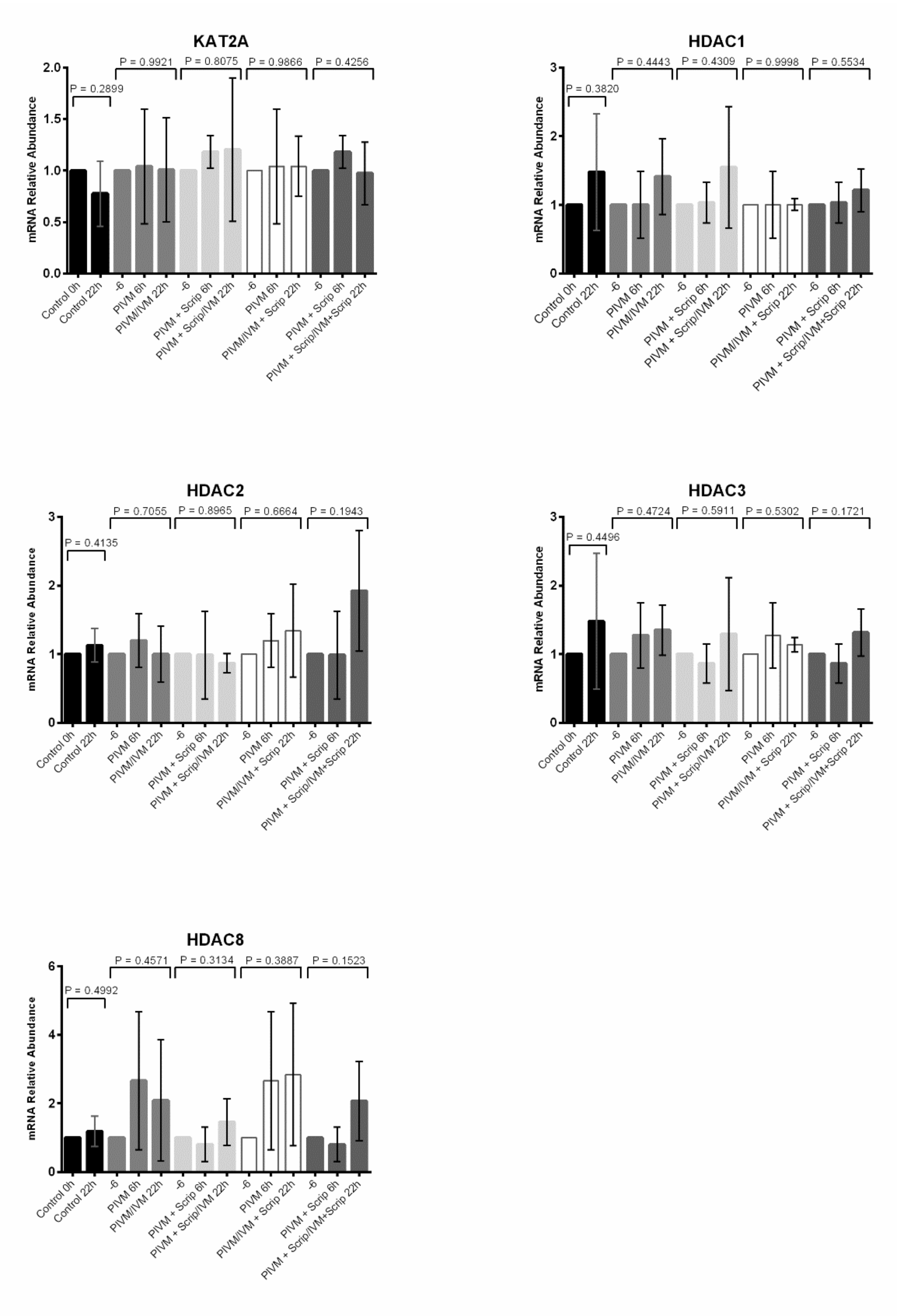

Figure S2: Transcripts levels of *KAT2A*, *HDAC1*, *HDAC2*, *HDAC3* and *HDAC8* quantified by RT-PCR of bovine 20 oocytes in different treatment groups. The data (mean ± SD) were normalized using the formula ΔΔCT (Pfaffl, 2001), and PPIA was the endogenous control. When the treatments were submitted to pre-maturation, we used the -6 legend to represent the control group 0 h. PIVM 6 h corresponds to oocytes that were submitted to pre-maturation for 6 h, PIVM + Scrip 6 h corresponds to oocytes submitted to pre-maturation with scriptaid for 6 h, PIVM / IVM 22 h corresponds to the oocytes that were pre-maturated and later matured in MIV medium for 22 h, PIVM + Scrip / 22 h IVM corresponded to oocytes submitted to pre-maturation with scriptaid and then matured for 22 h, PIVM + Scrip/IVM + Scrip corresponds to oocytes that underwent pre-maturation and maturation with addition of scriptaid.
