# Supplementary material for: Histone deacetylase inhibitor during in vitro maturation decreases developmental capacity of bovine oocytes
Source: PLoS One. 2021 Mar 5;16(3):e0247518. doi: 10.1371/journal.pone.0247518 (PMC7935280; doi:10.1371/journal.pone.0247518)
Supplement: S1 Table — (PDF) [file pone.0247518.s003.pdf]

Table S1. Effect of adding scriptaid during 6 and 22 hours of in vitro maturation (IVM) on cleavage at day 2 (D2) and blastocysts rates at D6 and D7 of development.

Treatment	N	Cleaved	D6	D7
Control	179	147(82,1%)	13(7,3%) [a]	50(27,9%) [a]
6h Scriptaid + IVM	175	136(77,7%)	3(1,7%) [b]	31(17,7%) [b]
22h Scriptaid + IVM	196	151(77%)	19(9,7%) [a]	60(30,6%) [a]

Parametric data were analyzed using the T test and non-parametric data were analyzed using the Mann Witney test at a significance level of 5%

[a, b] Different letters in the same column differ statistically from each other (P<0,05)
