# Supplementary material for: Histone deacetylase inhibitor during in vitro maturation decreases developmental capacity of bovine oocytes
Source: PLoS One. 2021 Mar 5;16(3):e0247518. doi: 10.1371/journal.pone.0247518 (PMC7935280; doi:10.1371/journal.pone.0247518)
Supplement: S2 Table — (PDF) [file pone.0247518.s004.pdf]

Table S2. Effect of scriptaid during pre-maturation and/or in vitro maturation on the proportion of cells of the internal cell mass relative to the amount of total cells (ICM / Total Cells)

Treatment	Total	0-20%	20-40%	40-60%
T1	39	1(2,6%)[b]	34(87,1%)[a]	4(10,3%)
T2	39	2(5,1%)[ab]	33(84,6%)[ab]	4(10,3%)
T3	32	3(9,4%)[ab]	27(84,4%)[ab]	2(6,3%)
T4	30	3(10%)[ab]	24(80%)[ab]	3(10%)
T5	30	5(16,7%)[a]	20(66,7%)[b]	5(16,7%)

[a,b,c] Different letters in the same column indicate significant Chi-Square difference ($P < 0.05$)

T1: Cumulus-oocyte complexes (COCs) submitted to maturation (IVM) for 22 hours
T2: COCs submitted to pre-maturation (PIVM) for 6 hours plus IVM for 22 hours
T3: COCs submitted to PIVM with scriptaid for 6 hours plus IVM 22 hours
T4: COCs submitted to PIVM plus IVM with Scriptaid for 22 hours
T5: COCs submitted to PIVM with scriptaid plus IVM with scriptaid for 22 hours
