# Supplementary material for: Histone deacetylase inhibitor during in vitro maturation decreases developmental capacity of bovine oocytes
Source: PLoS One. 2021 Mar 5;16(3):e0247518. doi: 10.1371/journal.pone.0247518 (PMC7935280; doi:10.1371/journal.pone.0247518)
Supplement: S3 Table — (PDF) [file pone.0247518.s005.pdf]

Table S3. Effect of scriptaid during pre-maturation (PIVM) of less competent oocytes (obtained from 1-3 mm follicles) on the amount of cells of the internal cell mass (ICM) and trophoectoderma (TE).

**Treatment**	**N**	**Total cells**	**TE**	**%TE**	**ICM**	**%ICM**
T1	19	131	88	70	35	30
T2	17	136	93	77	34	23
T3	21	122	82	62	35	38
T4	19	140	96	71	35	28
T5	7	115	79	70	46	30
T6	14	122	81	74	39	25

[a,b,c] Different letters in the same column indicate significant difference by Chi-Square test ($p < 0.05$)

T1: Cumulus-oocyte complexes (COCs) obtained from follicles of 3-8mm submitted to maturation (IVM) for 22 hours

T2: COCs obtained from follicles of 1-3mm submitted to IVM for 22 hours

T3: COCs obtained from of 3-8mm submitted to PIVM plus IVM 22 hours

T4: COCs submitted from of 3-8mm submitted to PIVM with scriptaid plus IVM 22 hours

T5: COCs submitted from of 1-3mm submitted to PIVM plus IVM 22 hours

T6: COCs submitted from of 1-3mm submitted to PIVM with scriptaid plus IVM 22 hours
